# Environmental Factors Influencing the Establishment of the Invasive Australian Redclaw Crayfish (*Cherax quadricarinatus*) in a Biosphere Reserve on the Central Mexican Plateau

**DOI:** 10.3390/life15040508

**Published:** 2025-03-21

**Authors:** Omar Y. Durán-Rodríguez, Daniel A. García-Ávila, J. Andrés Valencia-Espinosa, Eugenio Arroyo-Reséndiz, Martín J. Torres-Olvera, Juan P. Ramírez-Herrejón

**Affiliations:** 1 Institutional Doctoral Program in Biological Sciences, Facultad de Ciencias Naturales, Campus-Juriquilla, Universidad Autónoma de Querétaro, Av. de las Ciencias, Juriquilla, Santiago de Querétaro 76230, Querétaro State, Mexico; oduran22@alumnos.uaq.mx; 2 Institutional Master’s Program in Biological Sciences, Facultad de Ciencias Naturales, Campus-Juriquilla, Universidad Autónoma de Querétaro, Av. de las Ciencias, Juriquilla, Santiago de Querétaro 76230, Querétaro State, Mexico; dagarciabio@gmail.com; 3 Facultad de Ciencias Naturales, Campus-Juriquilla, Universidad Autónoma de Querétaro, Av. de las Ciencias, Juriquilla, Santiago de Querétaro 76230, Querétaro State, Mexico; jose.valencia@uaq.mx; 4 Escuela de Bachilleres, Concá Campus, Universidad Autónoma de Querétaro, Valle Agrícola, Concá, Arroyo Seco 76490, Querétaro State, Mexico; eugenio.arroyo@uaq.mx; 5 Escuela de Bachilleres, Jalpan Campus, Universidad Autónoma de Querétaro, Boulevard Policarpo Olvera, Fracc. El Coco, Jalpan de Serra 76490, Querétaro State, Mexico; 6 Water and Soil Quality Laboratory, SECIHTI-Universidad Autónoma de Querétaro, Carretera a Chichimequillas, Ejido Bolaños, Santiago de Querétaro 76140, Querétaro State, Mexico; ramirezherrejon@gmail.com

**Keywords:** crustacean, exotic species, environmental degradation, river, protected natural area

## Abstract

Crustaceans are among the most successful taxonomic groups in invasions worldwide. Humans can facilitate these invasions through introductions and disturbances in habitats. The Australian redclaw crayfish (*Cherax quadricarinatus*) is an invasive species with significant global ecosystem impacts. This species inhabits the Sierra Gorda Biosphere Reserve, in the Central Mexican Plateau. We hypothesize that environmental degradation facilitates the establishment and expansion of invasive crayfish. Seven sites along the Santa María River, within the reserve buffer zone, were assessed for seven months in 2023. We analyzed the abundance and density of the Australian redclaw crayfish in correlation with the environmental quality of the habitat. The results confirm that the establishment and spread of crayfish populations are related to water quality degradation and habitat alteration. The associated variables include increased total dissolved solids, greater substrate embedment, and degraded conditions on stream banks. Furthermore, the inverse relationship between the abundance of Australian redclaw crayfish and macroinvertebrate richness reinforces the hypothesis that more diverse native communities reduce the success of invaders. This study highlights the urgent need to implement management strategies focused on habitat restoration and the control of reproductive populations through the extirpation of mature individuals as critical measures for controlling the establishment and expansion of the invasive Australian redclaw crayfish.

## 1. Introduction

Invasive species pose a significant threat to biodiversity, leading to shifts in community structure, alterations to ecosystem functioning, and disruptions in the delivery of ecosystem services [1,2]. Their impact on ecosystem processes often arises from predation, parasitism, disease transmission, and habitat modification, while ecosystem services are affected by public health risks and economic damage [3,4]. Freshwater ecosystems are highly susceptible to anthropogenic disturbances that promote biological invasions [5,6,7], with the introduction of non-native species being one of the most critical threats to these habitats [8,9].

Human activities favor biological invasions, not only because of the extirpation of species from their native range and their introduction in new areas [6] but also because environmental degradation due to human activities can facilitate biological invasions. Disturbances typically lead to the reconfiguration and homogenization of available space (habitat) and resources, creating ’new vacancies’ in freshwater ecosystems for alien species with higher tolerance to environmental stressors and greater adaptability to altered conditions [10,11]. On the other hand, habitat complexity and heterogeneity can enhance freshwater ecosystems’ resistance to biological invasions by providing refuge for native fauna from predation or competition with invasive species [12].

Among freshwater invaders, crustaceans represent one of the most successful taxonomic groups of invasive species globally [13]. The Australian redclaw crayfish (*Cherax quadricarinatus* von Martens, 1868), a freshwater decapod crustacean, is native to Queensland and the Northern Territory of Australia, as well as southern Papua New Guinea [14,15]. This species has been introduced into more than 10 countries, including Indonesia, South Africa, Eswatini, China, and Zambezi Basin [16,17,18,19], for cultivation as an alternative food resource [20,21].

The Australian redclaw crayfish is considered a highly invasive species [22] that significantly impacts invaded ecosystems. It competes with native species for shelter and food [14,23,24,25]. Likewise, this species has a euryphagous feeding strategy that targets a wide range of aquatic organisms, including fish, amphibians, invertebrates, and aquatic plants [26]; thus, as generalist feeders, they can disrupt the ecosystem at all levels of the food web [27]. Additionally, the Australian redclaw crayfish is a potential vector for pathogens, including fungi, viruses, and bacteria, which pose a threat to native fauna [20,28,29,30,31,32].

The Sierra Gorda Biosphere Reserve (SGBR) stands out as one of Mexico’s most significant protected areas, hosting a wide variety of habitats that support diverse biotic communities, including both terrestrial and semi-aquatic vertebrates [33]. The SGBR is divided into different protection zones, such as buffer zones and core areas [34,35]. The core zones contain the most well-preserved ecosystems, which remain nearly pristine and provide refuge for species of flora and fauna that require special protection [34,35]. In the Santa Maria River and some of their affluents near a core zone of the SGBR, the Australian redclaw crayfish has recently been registered, representing a new threat to biodiversity in this biosphere reserve [36].

We hypothesize that environmental degradation, characterized by altered water quality and habitat disruption, facilitates the establishment and spread of the invasive redclaw crayfish (*Cherax quadricarinatus*) in the river ecosystem within the biosphere reserve. To test this hypothesis, we analyze the association between the density and abundance of redclaw crayfish and indicators of environmental degradation, including physicochemical water variables and indicators of river habitat conditions, biological integrity, and riparian quality. This knowledge is essential for understanding the Australian redclaw crayfish’s invasion dynamics and assessing and developing management strategies to reduce the spread and potential impacts within the SGBR and other regions.

## 2. Materials and Methods

The SGBR is a protected area in the Central Plateau of Mexico with an approximate extent of 383,500 ha. The SGBR occupies most of the Sierra Gorda, which is part of the Sierra Madre Oriental and covers the northern half of the state of Querétaro, the west of the state of Guanajuato, and a small portion of the state of San Luis Potosí. The SGBR belongs to the Pánuco River Hydrologic Region, which has two basins: Moctezuma River Basin, which includes the Moctezuma River, Extóraz River, and Axtla River, and Tamuín River Basin, which includes the Tampaón or Tamuín River, Santa María River, Verde River, and La Tinaja River [33,35].

**Figure 1:**
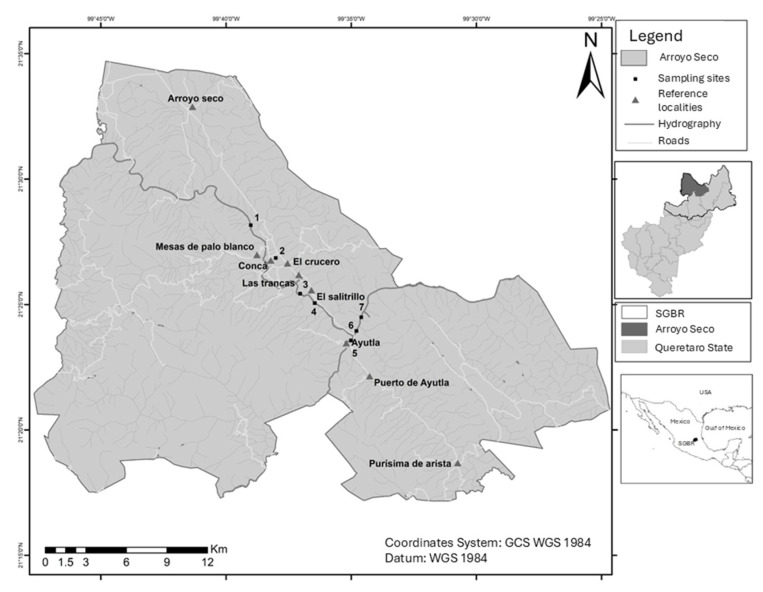
Geographic location of study area and study sites. SGBR = Sierra Gorda Biosphere Reserve; 1 = Puente de las Mesas; 2 = Concá Manantiales; 3 = El Higueron; 4 = El Salitrillo; 5 = Ayutla; 6 = Downstream of Adjuntas; 7 = Downstream of Jalpan River.

We sampled seven sites: five along the Santa María River, one at Concá Spring, and another on the Ayutla River. The latter two water bodies are both tributaries of the Santa María River, which forms the main course of the Tamuín Sub-basin. All of these sites are located in the Arroyo Seco municipality, in Queretaro State, in the SGBR (Figure 1). These sites are located within the SGBR buffer zone, less than 10 km upriver from one of the core zones of the reserve. The seven sampling sites within the Sierra Gorda Biosphere Reserve represent a gradient of environmental conditions, ranging from minimally impacted natural areas to highly disturbed habitats. Sites such as Ayutla (AYU), Puente de las Mesas (PM), and Downstream of Jalpan River (DRJ) exhibit well-preserved riparian corridors with diverse native vegetation, minimal human impact, and substrates dominated by larger particles like megalithal and macrolithal materials, providing a complex habitat structure. In contrast, sites like Concá Manantiales (CM) and El Salitrillo (SAL) are highly impacted by human activities, including tourism and sand dredging, with severely altered riparian zones, reduced vegetation cover, and substrates dominated by fine sediments (e.g., psammal and pelal), reflecting degraded habitat conditions. Intermediate conditions are observed at sites like El Higuerón (HIG) and Downstream of Adjuntas (DA), where moderate human impacts coexist with partially fragmented riparian vegetation and a mixed substrate composition. These environmental differences allowed for the assessment of the Australian redclaw crayfish distribution across varying habitat conditions (Table 1).

Australian redclaw crayfish specimens were collected using funnel traps with a single cone-shaped inlet (dimensions: 450 mm height, 300 mm diameter; mesh size: 5 mm) baited with fish. We set six traps at each site, with three placed in areas with current and three in pool zones. After 24 h, we collected the captured organisms. Sampling was conducted monthly in April, June, July, August, September, October, and November of 2023. Captured specimens of Australian redclaw crayfish were euthanized by placing them in slurry ice and then preserved in an 80% ethyl alcohol solution. Native crustaceans (i.e., *Macrobrachium* sp.) were measured, weighed, and released.

The identification of the specimens was determined using the diagnosis described by Arias-Rodríguez and Torralba-Burrial (2021) [37]. All specimens were weighed, measured, and sexed before preservation in a solution of 80% alcohol. The total length (TL) and carapace length (CL) were measured. The crayfish were placed on filter paper for several minutes to remove excess water and then weighed to the nearest 0.01 g, and we measured the total length (TL) and carapace length (CL) with calipers to the nearest 0.01 cm, following Rodriguez et al. (2014) and Sedik et al. (2019) [38,39]. The density of *Cherax quadricarinatus* was estimated using two complementary approaches: Global Density and Average Density. Global Density represents the total number of individuals captured per trap-day across the entire sampling period (7 months). Considering that six traps were deployed per site during each sampling event and each sampling lasted 24 h, the total trapping effort per site was 42 trap-days (6 traps × 7 sampling events). It was calculated as follows: total abundance (all sampling events)/42 trap-days. This metric reflects the overall capture rate standardized per day of trapping. Average Density refers to the mean density per sampling event by sampling. It was calculated by dividing the number of crayfish captured during each 24 h sampling event by the 42 trap-days used, then averaging these values across the seven sampling events at each site. The formula was as follows: average (abundance per sampling event/42 trap-days). This provides an estimation of the average number of crayfish captured per day.

Spawning-capable individuals were identified based on a combination of morphological and reproductive characteristics. Females carrying visible eggs were directly classified as spawning-capable. For individuals without visible eggs, maturity was assessed using weight thresholds and the presence of secondary sexual characteristics. Juveniles were defined as individuals weighing between 6 and 49 g, following commercial aquaculture classifications [40], while individuals weighing approximately 90 g or more were considered mature, consistent with the sexual maturity range reported for *Cherax quadricarinatus* (56.69–85.04 g) [41]. In males, secondary sexual characteristics included the presence of a distinctive red soft patch on the outer surface of the propodus of the claw, genital openings (gonopores) at the base of the fifth pereiopods, and the presence of the appendix masculina [42]. In females, maturity was confirmed by the presence of genital openings at the base of the third pereiopods. Additionally, intersex individuals, which may display mixed characteristics (e.g., one or two appendix masculina and both pairs of genital openings), were identified but categorized separately due to their ambiguous reproductive status [36]. This combination of morphological characteristics and weight thresholds allowed for reliable identification of spawning-capable individuals in the field.

We evaluated the physical, chemical, and biological conditions in April 2023 during the dry season when conditions were more stable [43]. This evaluation was conducted for each study site on a river section to standardize the sampling effort. The river section was equivalent to five times the river width, following the criteria of Mexican Standard NMX-AA-159-SCFI-2012 [44], which establishes the procedure for the determination of ecological flow in river basins. The physical condition of the habitat was assessed using a Visual-Based Habitat Assessment (VBHA) following the methodology of Barbour et al. (1999) [45], which includes 10 variables related to physical elements and processes, such as sinuosity, substrate and bank materials (epifaunal substrate/available cover, embeddedness, velocity/depth combinations), sediment retention areas (sediment deposition, channel flow status, channel alterations, frequency of riffles), riparian vegetation conditions (bank vegetative protection), riparian zone conditions (bank stability), and floodplain status (riparian vegetative zone width) (Table 2).

The condition of the riparian vegetation was evaluated following González del Tánago and García de Jalón (2011) [46], assessing qualitative attributes of the riverine zone of the river, including the space available for riparian functions (i.e., the longitudinal continuity of natural riparian vegetation, width dimension of the floodplain with riparian vegetation, and composition and structure of riparian vegetation variables) and indicators of the temporal evolution of the present structure (i.e., variables related to the natural regeneration of woody vegetation, bank conditions, and lateral connectivity, permeability, and soil profile conditions) (Table 2). These variables were scored on a numerical scale from 0 to 15 and added to provide a final habitat category. González del Tánago and García de Jalón (2011) [46] included descriptions of the characteristics and relative criteria to ensure consistency in the evaluation procedure, and the scores increased as the habitat quality increased. The actual riparian assessment process involved classifying the seven variables as very good (scored from 13 to 15), good (scored from 10 to 12), moderate (scored from 7 to 9), poor (scored from 4 to 6), or bad (scored from 1 to 3) based on the criteria described by González del Tánago and García de Jalón (2011) [46]. The data supporting the reported results are publicly archived on the Science Data Bank data storage platform, as stated in the Data Availability Statement.

We determined the chemical condition by measuring the water’s chemical and physical properties with a multimeter (Hach Hydromet Quanta, Loveland, CO, USA), including the pH, dissolved oxygen (mg/L), total dissolved solids (ppm), electrical conductivity (mS), and temperature (°C).

We assessed the biological condition by evaluating the index of biological integrity based on aquatic macroinvertebrate assemblages (IIBAMA) created by Pérez-Munguía and Pineda-López (2005) and validated by Torres-Olvera et al. (2018) [47,48]. This index includes six attributes that change with habitat degradation (here, we mention the attributes and ecological meanings taken directly from Torres-Olvera et al. (2018)) [48]. The attributes include the following: (I) The richness of taxa (RT) is limited by the heterogeneity of ecological process; high taxa richness can highlight habitat heterogeneity and is associated with an increased speciation likelihood. (II) Ephemeroptera, Plecoptera, and Trichoptera richness (EPT) is associated with the transformation of organic matter into available nutrients for superior trophic levels, and these groups are considered a good indicator of water quality. (III) The richness of sensitive insects (II) is included because aquatic insects can fly between freshwater ecosystems during adult stages as a survival strategy. The absence of sensitive insects is related to limiting conditions of temperature, dissolved oxygen, alkalinity, salinity, water flow rate, water level, and aquatic vegetation cover. For these reasons, sensitive insects offer current and long-term information about environmental conditions. (IV) The richness of sensitive taxa (IT) (not insects), which generally spend no part of their lifecycle out of the water, can indicate an ecosystem where the habitat quality has been optimal for a long time. (V) The mean tolerance value (MT) refers to the average value of tolerance of the sample. Tolerance represents the capability of aquatic macroinvertebrates to survive under environmental degradation. The values of tolerance show a relationship between anthropic stress and the presence of aquatic organisms in a spatiotemporal way. For this reason, they indicate the condition of freshwater systems. (VI) The number of fixed taxa to a substrate (FT) is moderately sensitive to water pollution and depends on biotope diversity and the heterogeneity of flow patterns. FT depletion can indicate the loss of aquatic habitat heterogeneity and availability, caused by the riverbank’s degradation. Also, land use changes in the catchment, which can increase fine sediment deposition, can reduce the available habitat and food resources for clinger organisms. The values of each attribute were used as response variables (not the value of the index) for data analyses (Table 2).

Macroinvertebrate samples were collected from all available habitats using a D-net (300 mm diameter, 300 μm mesh size) with a total sampling effort of 90 min. Macroinvertebrates were separated from detritus by placing the collected samples in a white tray, then picking the macroinvertebrates from the substrate with entomological tweezers. They were preserved directly in the field with 80% alcohol and transported to the Biotic Integrity Lab at UAQ-Campus Aeropuerto. Taxonomic identification of the macroinvertebrates was carried out at the family level using specialized keys (e.g., [49,50]).

The true diversity of aquatic macroinvertebrate assemblages was also estimated using the approach proposed by Jost (2006, 2007) through an assessment of the effective numbers of elements at the family level, which refers to the number of taxa equally probable and necessary to obtain a diversity value [51,52]. This method offers advantages over conventional indices, such as Shannon’s index, as it has mathematical properties that align more closely with what biologists intuitively expect from a diversity measure [52]. True diversity was estimated using the SPADE (2009) program. We calculated the first-order diversity to emphasize the influence of abundant species, given that aquatic macroinvertebrate communities often display a pattern of rare and abundant species, resulting in an uneven taxonomic distribution. The Jackknife estimator was employed. This estimator works by systematically recalculating diversity after removing one observation at a time, thereby generating an estimate that accounts for sample variability and rare taxa. It is well suited for macroinvertebrate assemblages due to its ability to reduce bias associated with under-sampling and provide more accurate estimates of true diversity [53,54].

We performed a principal component analysis (PCA) to elucidate patterns within the physical and chemical variables and identify the variables that explain the greatest variance with statistical significance [55]. Prior to the analysis, environmental variables were standardized by transforming them logarithmically to ensure comparability and prevent variables with larger scales from dominating the results. The PCA was based on a correlation matrix, which is suitable when variables are measured on different scales. Eigenvalues greater than five were considered to identify significant principal components. The PCA was conducted using data from all sampling sites to identify general patterns and key environmental gradients influencing the distribution of the Australian redclaw crayfish. A biplot was generated to illustrate the influence of environmental variables at each site and highlight the degradation gradient among them. The analysis included the pH, water temperature, electrical conductivity, total dissolved solids, dissolved oxygen, and index scores (IIBAMA, VBHA, and RQI). Additionally, we carried out a Spearman correlation analysis to examine the relationships between the abundance of the Australian redclaw crayfish and the environmental variables (physical, chemical, and biological) independently, in order to identify those most closely associated with species establishment. Additionally, we conducted a non-metric multidimensional scaling (NMDS) analysis to rank the data and identify patterns and associations between aquatic macroinvertebrate assemblages, the Australian redclaw crayfish, and environmental variables (including habitat, water quality, and community stability) [56]. These analyses were performed with PAST (version 3.07) software [57].

## 3. Results

A total of 760 Australian redclaw crayfish specimens were collected. The site with the highest abundance of this species was Concá Manantiales, a spring that feeds into the Santa Maria River. No individuals of the Australian redclaw crayfish were collected at the Ayutla site, a tributary of the Santa Maria River. Along the Santa Maria River, from upstream to downstream, 1 individual was collected at the upper site (Puente de las Mesas), 96 individuals at El Higuerón, 164 at El Salitrillo, 70 at Downstream of Adjuntas, and 12 at Downstream of Jalpan River (Table 3).

**Figure 2:**
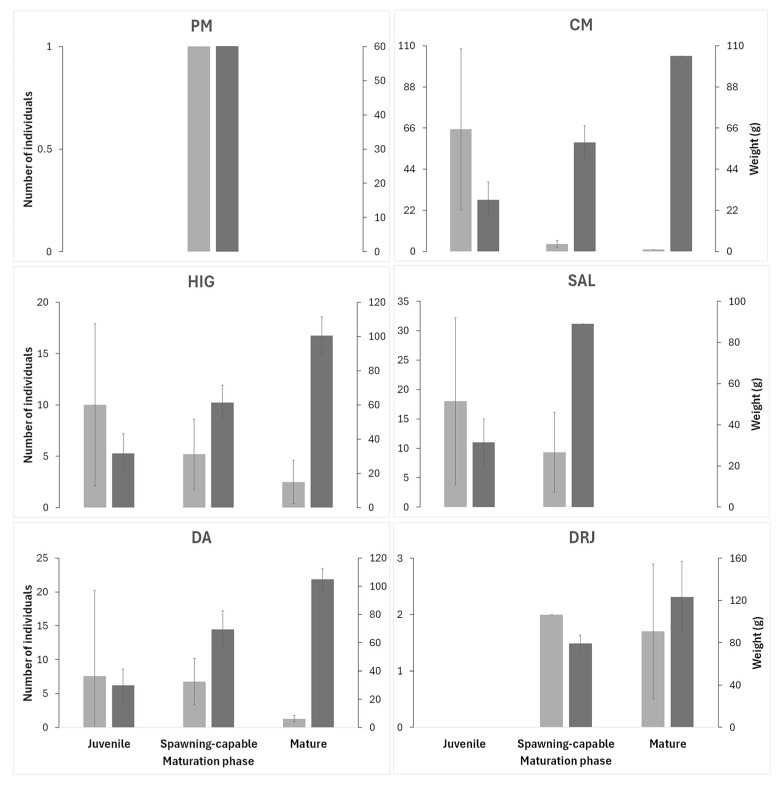
The number of individuals by maturation phase (light gray bar) and weight (dark gray bar) of the Australian redclaw crayfish (*Cherax quadricarinatus*) for each study site in the Sierra Gorda Biosphere Reserve, Central Mexican Plateau. PM = Puente de las Mesas; CM = Concá Manantiales; HIG = El Higuerón; SAL = El Salitrillo; DA = Downstream of Adjuntas; DRJ = Downstream of Jalpan River.

We found spawning-capable individuals at almost all study sites except Ayutla. We captured the largest and heaviest individuals at the most downstream site on the Santa María River (Downstream of Jalpan River), with an average weight of 103.64 g, including five mature individuals and four spawning-capable specimens; however, no juveniles were collected at this site (Figure 2). The site with the highest abundance of this species (Concá Manatiales) was dominated by juveniles (94%), with no mature individuals found. At Puente de las Mesas, the single individual collected was spawning-capable. In El Higuerón, 62.5% of individuals were juveniles, 32.3% were spawning-capable, and 5.2% were mature. At El Salitrillo, 65.6% were juveniles and 34.6% were spawning-capable. Downstream of Adjuntas, 54.3% were juveniles, 38.6% were spawning-capable, and 7.2% were mature (Figure 2).

**Figure 3:**
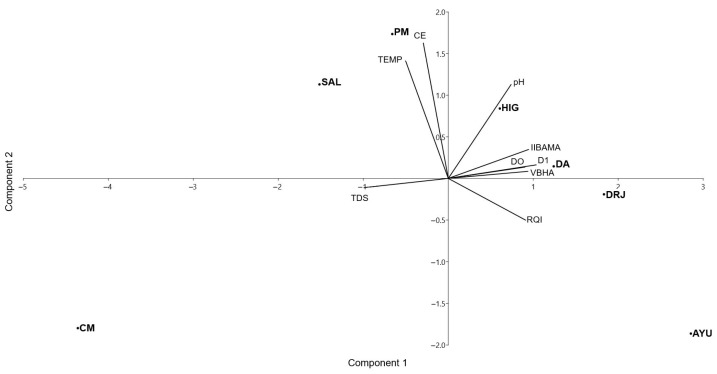
A principal component analysis biplot of environmental variables of sampling sites in the Sierra Gorda Biosphere Reserve, Central Mexican Plateau. SAL = El Salitrillo; PM = Puente de las Mesas; HIG = El Higuerón; DA = Downstream of Adjuntas; DRJ = Downstream of Jalpan River; AYU = Ayutla; CM = Concá Manantiales. TEMP = water temperature; CE = electrical conductivity; pH = hydrogen potential; IIBAMA = index of biological integrity based on aquatic macroinvertebrate assemblages; DO = dissolved oxygen; D1 = first-order alpha diversity; VBHA = Visual-Based Habitat Assessment; RQI = Riparian Quality Index; TDS = total dissolved solids.

The PCA indicated that the weight of the included variables was generally moderate and relatively balanced across components 1 and 2 (Table 4), which together explain 78% of the total variance in the set of environmental variables. As a result, the analysis did not provide elements for eliminating any environmental variables from the correlation with the abundance of the Australian redclaw crayfish. The variables with the highest coefficients in component 1 were the number of families of aquatic macroinvertebrates that live attached to the substrate (fixed taxa, FT) (0.2196), the true diversity of aquatic macroinvertebrate assemblages (D1) (0.2199), the number of families of aquatic macroinvertebrates intolerant to organic pollution (intolerant taxa, IT) (0.2178), the number of families of aquatic insects intolerant to organic pollution (intolerant insects, II) (0.2151), and the condition or stability of the stream banks (bank stability, BS) (0.2138). These variables represent biological and habitat quality factors related to the community structure and physical characteristics of the environment. The variables with the highest loadings in component 2 were the channel flow status (CFS) (0.3645), sediment deposition (SD) (0.3559), and the epifaunal substrate or available cover, which serves as refugia, feeding sites, or sites for spawning and nursery functions for aquatic macrofauna (epifaunal substrate, EPS) (0.3090). These variables are associated with the physical characteristics of the streambed and water flow, reflecting current conditions and the sediment structure. Collectively, these components capture the key environmental gradients influencing the distribution and establishment of the Australian redclaw crayfish in the study area. As shown in the PCA biplot of environmental variables, Ayutla (AYU) and Downstream of Jalpan River (DRJ) exhibit the lowest disturbance, followed by DA, HIG, PM, and SAL with intermediate values, while Conca Manantiales (CM) shows the highest disturbance (Figure 3).

The Spearman rank order correlations revealed some significant relationships between environmental variables and the abundance of the Australian redclaw crayfish (Table 5). The total dissolved solids (TDS) showed a strong positive correlation with Australian redclaw crayfish abundance (rs = 0.75, *p* = 0.0522), suggesting that higher levels of dissolved solids may favor the establishment of the species. The substrate embedment (EMB) was negatively correlated with abundance (rs = −0.7857, *p* = 0.0362), indicating that greater substrate embedment can favor the presence of the Australian redclaw crayfish due to the nature of the variable, since a higher rating of this variable showed a better condition or a lower degree of embedding. The taxon richness (RT) exhibited a perfect negative correlation (rs = −1, *p* < 0.0001), suggesting that more diverse communities are less likely to support a high abundance of the Australian redclaw crayfish. Although not statistically significant, bank stability (BS, rs = −0.7092, *p* = 0.0743) and the Visual-Based Habitat Assessment (VBHA, rs = −0.6786, *p* = 0.0938) also showed strong negative correlations, showing a potential trend where more stable and well-preserved habitats may reduce the success of the establishment of the Australian redclaw crayfish. These findings highlight the importance of water quality, biotic resistance, and habitat conditions in influencing the distribution and establishment of the Australian redclaw crayfish.

**Figure 4:**
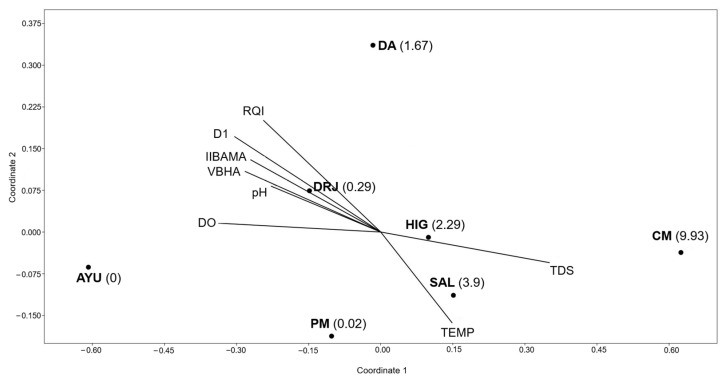
Non-metric multidimensional scaling ordination based on macroinvertebrate assemblages at the study sites in the Sierra Gorda Biosphere Reserve, Central Mexican Plateau, with environmental and biological variables as response variables. Values in parentheses indicate the density of the Australian redclaw crayfish. Stress: 0.06, r2 axis 1 = 0.75, r2 axis 2 = 0. AYU = Ayutla; DA = Downstream of Adjuntas; HIG = El Higuerón; SAL = El Salitrillo; CM = Concá Manantiales; PM = Puente de las Mesas; DRJ = Downstream of Jalpan River. DO = dissolved oxygen; pH = hydrogen potential; TEMP = water temperature; TDS = total dissolved solids; RQI = value obtained for the Riparian Quality Index; VBHA = value obtained in the Visual-Based Habitat Assessment; IIBAMA = value obtained for the index of biological integrity based on aquatic macroinvertebrates assemblages; D1 = first-order alpha diversity.

The non-metric multidimensional scaling (NMDS) analysis provided a clear ordination of sites based on environmental gradients (Figure 4). The first axis (r^2^ = 0.75) explained most of the variation, while the second axis (r^2^ = 0) did not contribute significantly. The environmental variables most strongly correlated with axis 1 were TDS (positively), DO, VBHA, IIBAMA, and pH (negatively), reflecting a gradient of water quality and habitat conditions influencing the distribution and density of the Australian redclaw crayfish. Concá Manantiales (CM) had a higher density and abundance of the Australian redclaw crayfish and showed a strong association with higher total dissolved solids (TDS). In contrast, the site Ayutla (AYU), with no presence of Australian redclaw crayfish, had the most negative score on axis 1, corresponding to higher dissolved oxygen (DO), lower temperatures (TEMP), and better habitat and integrity conditions (VBHA and IIBAMA). The sites Downstream of Jalpan River (DRJ) and Puente de las Mesas (PM) were positioned in the left zone closer to the center of the ordination, reflecting intermediate environmental conditions, and these sites showed the presence of the Australian redclaw crayfish but at a low density. On the other hand, the sites Downstream of Adjuntas (DA), El Salitrillo (SAL), and El Higuerón (HIG) were also closer to the center but on the right side of the ordination, reflecting more degraded conditions than DRJ and PM, and these sites showed higher densities of the Australian redclaw crayfish.

## 4. Discussion

This study demonstrated the successful establishment of the Australian redclaw crayfish (*Cherax quadricarinatus*) across multiple sites within the Santa María River and Concá Spring, in the Sierra Gorda Biosphere Reserve. The findings showed that the Australian redclaw crayfish is particularly abundant in degraded environments, so we cannot reject our hypothesis that environmental degradation, characterized by altered water quality and habitat disruption, facilitates the establishment and spread of this species in the river ecosystem. The environmental variables associated with higher densities of the Australian redclaw crayfish include increased total dissolved solids (TDS), greater substrate embedment, and degraded conditions of the stream banks, suggesting that the Australian redclaw crayfish thrives in habitats where the water quality and structural habitat features are compromised. These results contribute to identifying the environmental factors that facilitate the establishment and proliferation of invasive crayfish species and provide important insights into the mechanisms driving biological invasions in freshwater ecosystems.

Sites with higher densities of the Australian redclaw crayfish, such as El Salitrillo and El Higuerón, presented degraded environmental conditions. These conditions were reflected in variables such as substrate embedment, high TDS levels, and degraded stream banks. This pattern is consistent with previous findings that invasive species, such as the snail *Melanoides tuberculata*, and fishes such as the twospot livebearer (*Pseudoxiphophorus bimaculatus*) and the common carp (*Cyprinus carpio*), thrive in disturbed or human-impacted habitats [58,59,60]. In particular, the presence of egg-bearing females of the Australian redclaw crayfish in degraded areas, as observed in Indonesia, confirms the successful establishment of populations in impacted environments [18]. The ability of the Australian redclaw crayfish to accumulate heavy metals, as shown in previous studies [61], probably explains its success in these degraded environments. The species appears to have efficient detoxification mechanisms, allowing it to survive in habitats with high concentrations of contaminants (e.g., metals). Additionally, it is a useful bioindicator of substrate contamination due to its capacity to accumulate metals in its hepatopancreas [61].

The adaptability of the Australian redclaw crayfish to various environmental conditions, such as fluctuations in pH, temperature, and dissolved oxygen levels, enhances its ability to establish itself in diverse habitats [62,63]. In our study, this adaptability is supported by the presence of the Australian redclaw crayfish in sites with different physicochemical conditions. Specifically, the species was found in habitats with a range of dissolved oxygen levels (ranging within 5.81–12.27 mg/L), pH values (7.31–8.42), and temperatures (26.4–37.4 °C). These findings suggest that the Australian redclaw crayfish can tolerate environmental variability, which likely contributes to its invasive potential. Moreover, its reproductive potential is bolstered by increased sperm production at temperatures above 27 °C [64], which may explain its high reproductive success in warmer waters (i.e., >28 °C) within the study area. This is of particular interest in the context of climate change as the general temperature increases of 2–4 °C projected under the IS92 scenario for central–eastern Mexico may increase the risk of Australian redclaw crayfish establishment.

The inverse relationship between the abundance of the Australian redclaw crayfish and the richness of aquatic macroinvertebrate families found in our study suggests that ecosystems with greater biodiversity may be more resistant to the establishment of invasive species. This pattern may align with the biotic resistance hypothesis, where diverse native communities reduce the success of invaders [65]. This inverse correlation can reflect that such environments are less suitable for native species, reducing biotic resistance and facilitating the establishment of invasive species. Additionally, higher macroinvertebrate diversity is often associated with better water quality and habitat conditions [66]. Moreover, sites with elevated levels of total dissolved solids (TDS) and habitat degradation, where macroinvertebrate diversity is reduced, appeared to support higher Australian redclaw crayfish densities. This pattern suggests that human-induced disturbances, such as pollution and sedimentation, create ecological conditions where invasive species can thrive due to reduced competition and ecological niches that invasive species are well suited to exploit (cf. [67]).

These findings can help predict the presence and abundance of the Australian redclaw crayfish and contribute to its management in biological invasions. The negative correlation observed between *Cherax quadricarinatus* density and macroinvertebrate diversity suggests that habitat degradation may play a key role in facilitating the establishment of this invasive species. This pattern likely reflects habitat quality rather than direct biotic interactions, such as resource competition. *Cherax destructor*, for example, thrives in eutrophic, canopy-free habitats where autochthonous resources dominate [68]. Similarly, *C. quadricarinatus* may benefit from the high productivity of eutrophic systems, where easily digestible resources are abundant. This is consistent with laboratory studies showing that the species achieves greater growth and survival rates when fed low-fiber diets, reflecting its limited capacity to digest structural plant material [69]. These findings suggest that the presence of *C. quadricarinatus* in degraded habitats may be linked to its ability to exploit high-productivity environments, which often have reduced macroinvertebrate diversity. Additionally, despite its flexible trophic niche, *C. quadricarinatus* tends to occupy lower trophic levels in the food web [70]. Abiotic factors such as total dissolved solids (TDS), substrate embedment, and shoreline degradation appear to be stronger predictors of *Cherax* abundance. However, macroinvertebrate richness may serve as an indirect indicator of habitat quality and should be further explored as a potential predictor of invasive species abundance.

Our findings align with studies on other invasive crayfish species, such as *Procambarus clarkii*, which also thrive in eutrophic and degraded habitats. In these conditions, eutrophication, riparian canopy removal, and lower flow velocities decrease habitat suitability for native species, thereby reducing biotic resistance and indirectly facilitating the establishment of invaders [71]. In such environments, other invasive crayfish like *Austropotamobius pallipes* and *Procambarus clarkii* can exploit altered ecological conditions such as increased availability of organic matter, reduced competition, and lower predation pressure, which may enhance their growth and establishment [72]. These species exhibit physiological and ecological plasticity, allowing them to tolerate fluctuating environmental conditions, including low dissolved oxygen levels and higher nutrient concentrations, which often limit native species survival and reproduction.

Although this study focused on the environmental conditions influencing the establishment of the Australian redclaw crayfish, the observed differences in population structure across sites suggest underlying population dynamics that require further investigation. Higher densities of the Australian redclaw crayfish were recorded in more disturbed sites; however, body size did not appear to be significantly affected by environmental degradation. The lower densities observed in less degraded sites, without compromising individual growth, suggest that the species can adapt to these environments, albeit at reduced population densities. Additionally, differences in the proportions of juveniles and adults were observed among sites, which may be related to ontogenetic dietary shifts. Juveniles, with higher protein requirements, tend to rely on zooplankton and small macroinvertebrates, which may favor their proliferation in areas with high organic matter loads and degraded habitat conditions [68,73]. This aligns with the higher proportion of juveniles recorded in more degraded sites, such as Concá Manantiales (CM) and El Salitrillo (SAL). In contrast, adults, whose diet is more dependent on detritus and macrophytes, may be better suited to a broader range of habitats, including less degraded sites where resource diversity and habitat complexity are greater [73]. While these patterns provide valuable insights, it is premature to draw definitive conclusions. Factors such as fishing pressure, predator presence, and specific environmental conditions could be more important for the population dynamics of the Australian redclaw crayfish in invaded ecosystems.

On the other hand, the impact of the Australian redclaw crayfish on native biodiversity could extend beyond competition for shelter and resources. The presence of invasive crayfish species is linked to a decline in benthic macroinvertebrate biodiversity in aquatic ecosystems [74], as we observed in sites with higher abundance of the Australian redclaw crayfish in our study. The population establishment of the Australian redclaw crayfish in degraded habitats of the Sierra Gorda Biosphere Reserve may lead to significant changes in the aquatic community structure, such as shifts in community composition, reductions in functional diversity, and alterations in trophic interactions, including the disruption of predator–prey dynamics and nutrient cycling processes, through mechanisms such as bioturbation and selective predation on native macroinvertebrates. Competition for resources and shelter with native crustaceans, as documented in other invasions, could reduce the abundance and diversity of these native species [75,76], potentially leading to local population declines or shifts in species dominance. Interestingly, our results showed that sites with higher macroinvertebrate diversity, such as Ayutla (AYU), had no Australian redclaw crayfish presence, while sites with lower diversity, like Concá Manatiales (CM) and El Salitrillo (SAL), supported higher densities of the species. This pattern suggests that reduced native biodiversity may facilitate the establishment of the Australian redclaw crayfish; in turn, its presence could exacerbate biodiversity loss, creating synergistic effects that allow its successful establishment. These ecological processes alter community structure and ecosystem processes, such as organic matter decomposition and nutrient cycling [77].

The Australian redclaw crayfish threatens the integrity of the aquatic ecosystems in the Sierra Gorda Biosphere Reserve and poses a risk to native species, particularly *Macrobrachium* sp., a native crayfish in the same river systems and habitats. The presence of the invasive Australian redclaw crayfish increases the risk of competitive displacement of *Macrobrachium* sp., which is already facing pressure from habitat degradation. The Australian redclaw crayfish is known for its aggressive behavior and ability to outcompete native species for essential resources (e.g., macrophytes and detritus) [78]. This competitive pressure may lead to reduced access to food and shelter for *Macrobrachium* sp. While *Macrobrachium* sp. is a robust species, with adult specimens possessing physical strength and resistance that may allow them to defend against aggressive interactions with the Australian redclaw crayfish, juvenile *Macrobrachium* sp. are more vulnerable due to their smaller size, making them more susceptible to predation or displacement by mature Australian redclaw crayfish.

The presence of the Australian redclaw crayfish can exacerbate habitat degradation, which can directly affect *Macrobrachium* sp. and other native species. Invasive crayfish species are known to alter the physical structure of the environment by burrowing and disturbing the substrate, leading to increased turbidity and sedimentation [71]. These changes can degrade the quality of habitats that are critical for the survival of native species, particularly in areas with already lower water quality and limited shelter. The loss of critical habitats and increased predation pressure by the invasive Australian redclaw crayfish can lead to the decline of native populations.

As Rodríguez-Cruz et al. (2023) [36] emphasized, understanding the distribution and invasion dynamics of the Australian redclaw crayfish is crucial for developing effective management strategies to mitigate its spread and impact within the Sierra Gorda Biosphere Reserve. Restoring habitats, replanting riparian vegetation, improving water quality by reducing pollution and eutrophication, and managing local dredging could reduce the invasion success. Additionally, managing reproductive populations (egg-bearing females, mature individuals, and intersex males) may be a key action to limiting the population’s success. This management could involve targeted removal efforts focused on these reproductive individuals during breeding seasons, such as the use of baited traps designed to capture larger, mature crayfish. By reducing the reproductive potential of the population, this strategy can decrease recruitment rates and slow the spread of the Australian redclaw crayfish in invaded ecosystems [16,79]. However, it is essential to consider the reproductive characteristics associated with crayfish, which could represent a challenge for the success of management strategies. On the one hand, crayfish have different reproductive morphotypes that are essential for understanding the fine reproductive period [80,81]. On the other hand, it is necessary to locate females and intersexual individuals who tend to act like males, since these are the ones who tend to have a higher proportion of females in their litter [6]. The capture of these organisms is recommended as part of a control plan, especially at the beginning of invasions, to reduce propagule pressure [79].

This study provides the principal environmental factors that facilitate the establishment and proliferation of the invasive Australian redclaw crayfish, including elevated levels of total dissolved solids (TDS), reduced substrate embedment, lower macroinvertebrate taxon richness, and degraded habitat conditions, such as poor bank stability and reduced riparian vegetation cover; however, the relatively short sampling period may not capture the population dynamics of the species. Additionally, some biotic factors that could influence the distribution of the species, such as the presence of predators or competitors, were not considered. Future studies should address these variables to provide a more comprehensive understanding of the relationships between the Australian redclaw crayfish and local biodiversity. We suggest long-term studies to assess how the Australian redclaw crayfish populations evolve in response to habitat improvements. Experimental research evaluating the effectiveness of different management strategies, such as species removal or riparian restoration, could provide valuable insights for controlling this invasion and understanding the invasion dynamics. It is also significant to monitor the long-term effects on native biodiversity and main ecosystem processes, such as primary productivity and organic matter decomposition. There is an urgent need for further studies and management measures for improving habitat quality and limiting the spread of this species to protect native biodiversity.

## Figures and Tables

**Table 1:** Habitat description of sampling sites in Sierra Gorda Biosphere Reserve, Central Mexican Plateau. Sites: PM = Puente de las Mesas; HIG = El Higueron; SAL = El Salitrillo; DA = Downstream of Adjuntas; DRJ = Downstream of Jalpan River; CM = Concá Manantiales; AYU = Ayutla. Substrate size: megalithal (>40 cm); macrolithal (20–40 cm); mesolithal (6–20 cm); microlithal (2–6 cm); akal (small gravel); psammal (sand); pelal (slit, sludge). Habitat description: HMU = hydromorphological units; HS = hydraulic signature. Characterization of hydraulic signature: shallow (<0.5 m), wading (0.5–1 m), deep (>1 m); slow (<25 cm/s), flows (25–50 cm/s), fast (>50 cm/s).

Site	Shore Line	Riparian Vegetation	Substrate	Habitat	Main Impacts
PM	Banks significantly modified by human action.	Average width of riparian corridor significantly altered by human action. Riparian vegetation appears in small patches covering less than 30% of segment length.	Particle stratification that provides diversity of niche space. Akal (60%), psammal (20%), macrolithal (10%), mesolithal (5%), megalithal (3%), microlithal (2%).	Overhanging vegetation, canopy cover shading (3%), submerged vegetation, woody debris, undercut banks, and boulders. HMU: glide (80%), ruffle (10%), run (5%), and backwater (5%). HS: shallow fast (75%), wading fast (10%), shallow slow (5%), wading flows (5%), shallow flows (3%), and shallow slow (2%).	Natural area with moderate human impact (road, livestock, and tourism).
HIG	Banks moderately modified by human action in their form and processes.	Average width of riparian corridors significantly reduced by human action, with average width less than 1 active channel width. Riparian corridor moderately fragmented with 50% of natural coverage including several strata.	Particles (25–30%) surrounded by fine sediment. Mesolithal (40%), psammal (40%), microlithal (10%), akal (10%).	Overhanging vegetation, canopy cover shading (3%), shallow margins, undercut banks, boulders, and woody debris. HMU: pool (40%), run (40%), ruffle (10%), and backwater (10%) HS: wading flows (40%), deep slow (40%), shallow fast (10%), and shallow slow (10%).	Natural area with minimal human impact.
SAL	Banks severely altered by human action. Channel margins connected to urbanized areas and roads.	Average width of riparian corridors severely reduced due to human action. Few riparian woody species; herbaceous communities predominate due to human actions.	Particles (>75%) surrounded by fine sediment. Psammal (90%), mesolithal (5%), macrolithal (5%).	Overhanging vegetation, canopy cover shading (2%), undercut banks, and woody debris. HMU: glide (95%) and ruffle (5%). HS: shallow slow (90%), shallow flows (5%), and shallow fast (5%).	Area impacted by sand dredging and roads.
DA	Banks moderately modified by human action in their form and processes.	Moderately restricted by human action. Average width of around 3 times active channel width. Riparian corridors moderately fragmented with 60% of natural coverage including several strata.	Particles (30–40%) surrounded by fine sediment. Megalithal (70%), psammal (20%), macrolithal (10%).	Overhanging vegetation, canopy cover shading (5%) boulders, and shallow margins. HMU: run (50%), rapid (25%), pool (20%), and backwater (5%). HS: wading fast (40%), deep fast (25%), deep flows (15%), wading flows (15%), and shallow slow (5%).	Natural area with moderate human impact (tourism, livestock).
DRJ	Banks moderately modified by human action in their form and processes.	Continuity and coverage of riparian corridor in natural conditions including a mix of species corresponding to native vegetation associations of river segment, with different strata.	Particle stratification that provides diversity of niche space. Mesolithal (70%), macrolithal (20%), pelal (10%).	Overhanging vegetation, canopy cover shading (50%), shallow margin both left and right, submerged vegetation, undercut banks, and boulders. HMU: run (60%), rapid (20%), pool (10%), and fast run (10%). HS: wading flows (60%), shallow fast (20%), wading fast (10%), deep slow (5%), and deep flows (5%).	Natural area with minimal human impact.
CM	Banks severely altered by human action.	Average width of riparian corridor significantly altered by human action. Riparian vegetation is reduced to isolated trees or shrubs, leaving large open areas), including only one stratum.	Particles (>75%) surrounded by fine sediment. Microlithal (50%), debris (20%), pelal (15%), psammal (5%), akal (5%), mesolithal (5%).	Overhanging vegetation, canopy cover shading (70%), undercut banks, and submerged vegetation. HMU: pool (55%), glide (40%), and riffle (5%). HS: wading slow (55%), wading flows (40%), and shallow fast (5%).	Highly impacted by tourism activities.
AYU	Banks moderately modified by human action in their form and processes.	Moderately restricted by human action. In valley surrounded by vegetation.	Particle stratification that provides diversity of niche space. Megalithal (60%), macrolithal (20%), mesolithal (15%), microlithal (5%).	Overhanging vegetation, canopy cover shading (20%), shallow margin left, boulders, and woody debris. HMU: glide (60%), pool (20%), rapid (10%), and run (10%). HS: wading flows (60%), wading slow (20%), wading fast (10%), and deep slow (10%).	Natural area with moderate human impact (tourism).

**Table 2:** Environmental variables of sampling sites in the Sierra Gorda Biosphere Reserve, Central Mexican Plateau. For variable values, the RQI (values of 1–150) is the sum of the variables RW*, LC*, CS*, ADR, BC, LAC, and VC. Each variable is evaluated on a scale of “1 to 15”, where “1” represents bad quality and “15” very good quality. For variables marked with “*”, each margin is assessed separately, representing the condition of each side, and their combined score reflects the total score (values of 1–30). The VBHA (values of 1–200) is the sum of the variables EPS, EMB, V/D, SD, CFS, CA, FR, BS*, and VP*, and is equivalent to RW*. Additionally, it includes the score of another variable (riparian width), which is not included to avoid redundancy. Each variable is evaluated on a scale of “0 to 20”, where “0” represents the poorest or most altered condition of the variable and “20” the most optimal or healthy condition. For variables marked with “*”, each margin is evaluated separately (values of 1–10), and their combined score reflects the total score (values of 1–20). The IIBAMA (values of 0–24) is the sum of the variables RT, EPT, II, IT, MT, and FT. The resulting value of each variable is assigned a value between 1 and 4, depending on the point category.

Index	Variable	Acronym	Variable Description
RQI Riparian Quality Index (González-del-Tánago and García-de-Jalón, 2011) [46] An index developed to assess and characterize the ecological status of riparian systems.	Riparian width	RW *	Assesses restrictions to the riparian corridor caused by human influence. When there are no restrictions, the riparian width has its natural borders, and vegetation covers all land that is between the channel and adjacent slopes.
	Longitudinal continuity	LC *	An estimation of the intensity of fragmentation of the riparian vegetated area based on the size and frequency of open areas created by human actions (i.e., land-use).
	Composition and structure of riparian vegetation	CS *	This variable helps assess the condition of riparian composition by evaluating the vegetation’s natural succession stages.
	Age diversity and regeneration	ADR	This variable refers to the age classes of woody species in the riparian zone. It helps to evaluate the regeneration of woody species.
	Bank condition	BC	This variable helps to assess the heterogeneity of the water shoreline, stability of banks, and changes in erosion and sedimentation.
	Lateral connectivity	LAC	The variable assesses how much the flow regulation has been altered by morphological changes in the margins of the river or by channelization works that prevent the occurrence of natural bank flooding.
	Vertical connectivity	VC	The level of alterations to the soil surface that reduce natural infiltration and alterations to substrata that reduce alluvial permeability, subsurface flows, and groundwater connectivity.
VBHA Visual-Based Habitat Assessment (Barbour et al., 1999) [45] A qualitative index to visually evaluate the environmental condition of rivers and streams by assessing their physical and ecological characteristics.	Epifaunal substrate	EPS	The abundance and diversity of submerged structures in a stream (such as cobbles, rocks, logs, and undercut banks) that shape habitat complexity. These features provide refuge, feeding grounds, and spawning sites for macrofauna.
	Substrate embedment	EMB	The degree to which rocks and snags are buried in streambed sediments. Higher embeddedness reduces the surface area for shelter, spawning, and egg incubation.
	Velocity and depth regime variations	V/D	The presence of diverse flow patterns (slow–deep, slow–shallow, fast–deep, and fast–shallow) enhancing habitat complexity.
	Sediment deposition	SD	The accumulation of sediment in pools and the alteration of river bottoms. Excessive deposition indicates instability and reduces habitat suitability.
	Channel flow status	CFS	Refers to the degree to which the stream channel is filled with water. Changes in flow, caused by factors such as channel widening or flow reduction, limit suitable habitats for aquatic organisms.
	Channel alteration	CA	Refers to significant changes in the stream’s shape, often due to human activities like straightening, deepening, or diverting for flood control or irrigation.
	Riffle frequency	FR	Measures the occurrence of riffles, which contribute to habitat diversity and fauna richness. More frequent riffles enhance habitat quality.
	Bank stability	BS *	Assesses the degree of bank erosion and potential for collapse. Steep, unstable banks with crumbling soil, exposed roots, or lack of vegetation indicate sediment movement issues and reduced habitat quality.
	Vegetation protection	VP *	Refers to the extent of vegetation on stream banks and the adjacent riparian zone. The presence of native vs. exotic plants and the impact of grazing or urbanization on vegetation are also considered.
IIBAMA Index of biological integrity based on aquatic macroinvertebrate assemblages (Pérez-Munguía and Pineda-López, 2005; Torres-Olvera, et al., 2018) [47,48] This index was developed to estimate the environmental condition of rivers and streams in central México. The index is based on families of aquatic macroinvertebrates as indicators of degradation in river ecosystems.	Taxon richness	RT	The number of aquatic macroinvertebrate families in a sample. Higher taxa richness may indicate habitat heterogeneity, which is related to refuge availability and an increased speciation likelihood.
	Ephemeroptera, Plecoptera, and Trichoptera richness	EPT	Families in these Orders (excluding Baetidae) are associated with the transformation of organic matter into nutrients and are sensitive to environmental stress.
	Intolerant insects	II	Aquatic insect families that are sensitive to environmental degradation. The absence of sensitive insects is an indicator of alterations in environmental conditions (i.e., dissolved oxygen, temperature, water level).
	Intolerant taxa	IT	Refers to variables such as variable II plus other taxa of macroinvertebrates that are not tolerant.
	Mean tolerance	MT	Corresponds to the average of the tolerance values present in the sample.
	Fixed taxa	FT	Corresponds to the number of taxa that have life habits fixed to the substrate.

**Table 3:** The number of individuals, relative abundance, and density (Global Density = total abundance (all sampling events)/42 trap-days, Average Density = average (abundance per sampling event/42 trap-days)) of the Australian redclaw crayfish (*Cherax quadricarinatus*) by site in the Sierra Gorda Biosphere Reserve, Central Mexican Plateau. NI = number of individuals; RA = relative abundance; AYU = Ayutla; DA = Downstream of Adjuntas; HIG = El Higuerón; SAL = El Salitrillo; CM = Concá Manantiales; PM = Puente de las Mesas; DRJ = Downstream of Jalpan River.

Study Site	NI	RA	Global Density	Average Density
AYU	0	0	0	0
DA	10 ± 14.3	5.7	1.67	0.24 ± 0.3
HIG	13.7 ± 12.5	9.4	2.29	0.33 ± 0.29
SAL	23.4 ± 19.6	24.5	3.9	0.56 ± 0.47
CM	59.6 ± 47.5	58.5	9.92	1.4 ± 1.1
PM	0.14 ± 0.38	0	0.02	0.003 ± 0.009
DRJ	1.7 ± 1.4	1.9	0.29	0.04 ± 0.03

**Table 4:** A principal component analysis of environmental variables of sampling sites in the Sierra Gorda Biosphere Reserve, Central Mexican Plateau. pH = hydrogen potential; CE = electrical conductivity; TEMP = water temperature; TDS = total dissolved solids; DO = dissolved oxygen; EPS = epifaunal substrate; EMB = substrate embedment; V/D = velocity and depth regime variations; SD = sediment deposition; CFS = channel flow status; CA = channel alteration; FR = riffle frequency; BS = bank stability; VP = vegetation protection; VBHA = Visual-Based Habitat Assessment; RW = riparian width; LC = longitudinal continuity; CS = composition and structure; ADR = age diversity and regeneration; BC = bank condition; LAC = lateral connectivity; VC = vertical connectivity; RQI = Riparian Quality Index; RT = taxon richness; EPT = Ephemeroptera, Plecoptera, and Trichoptera richness; II = intolerant insects; IT = intolerant taxa; MT = mean tolerance; FT = fixed taxa; IIBAMA = index of biological integrity based on aquatic macroinvertebrate assemblages; D1= first-order alpha diversity.

	PC 1	PC 2
Eigenvalue	19.5955	6.3525
Percentage variance	59.38	19.25
pH	0.1775	−0.2208
CE	−0.0199	−0.3479
TEMP	−0.0798	−0.2378
TDS	−0.2030	−0.0157
DO	0.1806	−0.0281
EPS	0.0641	0.3090
EMB	0.1593	0.1572
V/D	0.2079	−0.0577
SD	−0.0067	0.3559
CFS	0.0278	0.3645
CA	0.2098	−0.1047
FR	0.2134	−0.0821
BS	0.2138	0.0690
VP	0.0628	0.1302
VBHA	0.2075	0.1048
RW	0.1485	0.1704
LC	0.1423	0.1356
CS	0.1445	0.1773
ADR	0.1752	−0.2115
BC	0.2030	0.0774
LAC	0.1214	−0.2405
VC	0.1996	0.0009
RQI	0.1947	0.1637
RT	0.1841	0.1378
EPT	0.1857	−0.1912
II	0.2151	−0.0181
IT	0.2178	0.0024
MT	−0.1757	0.2107
FT	0.2196	−0.0109
IIBAMA	0.2132	0.0313
D1	0.2199	0.0157

**Table 5:** Spearman correlations between the abundance of Australian redclaw crayfish (*Cherax quadricarinatus*) and environmental and biological variables in the Sierra Gorda Biosphere Reserve, Central Mexican Plateau. rs = Spearman rank order correlation; pH = hydrogen potential; CE = electrical conductivity; TEMP = water temperature; TDS = total dissolved solids; DO = dissolved oxygen; EPS = epifaunal substrate; EMB = substrate embedment; V/D = velocity and depth regime variations; SD = sediment deposition; CFS = channel flow status; CA = channel alteration; FR = riffle frequency; BS = bank stability; VP = vegetation protection; VBHA = Visual-Based Habitat Assessment; RW = riparian width; LC = longitudinal continuity; CS = composition and structure; ADR = age diversity and regeneration; BC = bank condition; LAC = lateral connectivity; VC = vertical connectivity; RQI = Riparian Quality Index; RT = taxon richness; EPT = Ephemeroptera, Plecoptera, and Trichoptera richness; II = intolerant insects; IT = intolerant taxa; MT = mean tolerance; FT = fixed taxa; IIBAMA = index of biological integrity based on aquatic macroinvertebrate assemblages; D1 = first-order alpha diversity. The superscript “a” next to the *p* -values indicates that the marked variables are statistically significant at the established significance level (*p* < 0.05).

Variable	rs	*p*
pH	−0.0541	0.9084
CE	0.2143	0.6445
TEMP	0.3214	0.4821
TDS	0.75	0.0522
DO	−0.3929	0.3833
EPS	−0.6667	0.1019
EMB	−0.7857	0.0362 ^a^
V/D	−0.4364	0.3276
SD	−0.1081	0.8175
CFS	−0.3368	0.4601
CA	−0.5455	0.2053
FR	−0.393	0.3832
BS	−0.7092	0.0743
VP	−0.4491	0.3121
VBHA	−0.6786	0.0938
RW	−0.5455	0.2053
LC	−0.3143	0.5441
CS	−0.4505	0.3104
ADR	0.0741	0.8745
BC	−0.393	0.3832
LAC	0	1
VC	−0.2594	0.5742
RQI	−0.7027	0.0782
RT	−1	<0.0001 ^a^
EPT	0	1
II	−0.4364	0.3276
IT	−0.593	0.1605
MT	0	1
FT	−0.6365	0.1243
IIBAMA	−0.4546	0.3054
D1	−0.70273	0.089683

## Data Availability

The data supporting the reported results are publicly archived on the Science Data Bank data storage platform, which can be accessed through the following link: https://doi.org/10.57760/sciencedb.14208.

## Abbreviations

The following abbreviations are used in this manuscript:

SGBR	Sierra Gorda Biosphere Reserve
TL	Total length
CL	Carapace length
VBHA	Visual-Based Habitat Assessment
PCA	Principal component analysis
NMDS	Non-metric multidimensional scaling
PC	Principal component
DO	Dissolved oxygen
TDS	Total dissolved solids
TEMP	Water temperature
CE	Electrical conductivity
EPS	Epifaunal substrate
EMB	Substrate embedment
V/D	Velocity and depth regime variations
SD	Sediment deposition
CFS	Channel flow status
CA	Channel alteration
FR	Riffle frequency
BS	Bank stability
VP	Vegetation protection
RW	Riparian width
LC	Longitudinal continuity
CS	Composition and structure
RQI	Riparian Quality Index
ADR	Age diversity and regeneration
BC	Bank condition
LAC	Lateral connectivity
VC	Vertical connectivity
RT	Taxon richness
EPT	Ephemeroptera, Plecoptera, and Trichoptera richness
II	Intolerant insects
IT	Intolerant taxa
FT	Fixed taxa
MT	Mean tolerance
IIBAMA	Index of biological integrity based on aquatic macroinvertebrate assemblages
D1	First-order alpha diversity
NI	Number of individuals
RA	Relative abundance
AYU	Ayutla
DA	Downstream of Adjuntas
HIG	El Higuerón
SAL	El Salitrillo
CM	Concá Manantiales
PM	Puente de las Mesas
DRJ	Downstream of Jalpan River
Rs	Spearman rank order correlation
